# A subset of microRNAs defining the side population of a human malignant mesothelioma cell line

**DOI:** 10.18632/oncotarget.17086

**Published:** 2017-04-13

**Authors:** Myung-Chul Kim, Na-Yon Kim, Yu-Ri Seo, Yongbaek Kim

**Affiliations:** ^1^ Laboratory of Clinical Pathology, College of Veterinary Medicine, Seoul National University, Gwanak-Gu, Seoul 151-742, The Republic of Korea; ^2^ BK21 PLUS Program for Creative Veterinary Science Research, College of Veterinary Medicine, Seoul National University, Gwanak-Gu, Seoul 151-742, The Republic of Korea; ^3^ Research Institute for Veterinary Science, College of Veterinary Medicine, Seoul National University, Gwanak-Gu, Seoul 151-742, The Republic of Korea

**Keywords:** mesothelioma, side population, microRNA, intratumoral heterogeneity, microarray

## Abstract

This study was performed to investigate the global expression profile of microRNAs in distinct subpopulations of a human malignant mesothelioma cell line. Total RNAs were isolated from the sorted side population and non-side population of MS1. The RNAs were subjected to analysis using Affymetrix GeneChip microRNA Arrays. After data extraction and normalization, a subset of microRNAs defining cell subpopulations was identified using bioinformatics softwares. Based on the criteria of 2-fold difference and the p-value of < 0.05, a total of 95 microRNAs were differentially expressed in the side population compared to the non-side population. Functional ontology revealed that target genes of the miRNAs were categorized into various gene ontology terms, such as stem cell maintenance, cell proliferation, programmed cell death, cell migration, and cellular response to stress. The Kyoto Encyclopedia of Genes and Genomes analysis showed that ErbB-2 receptor tyrosine kinases signaling pathway was the most represented. Integrated analysis of MiRTarBase and RNA-seq identified 12 target genes of microRNAs defining side population, including *DDIT4* and *ROCK2*. The present study indicates that a distinct set of microRNAs may be critically involved in the generation and maintenance of heterogeneous subpopulations of cancer cells. They could be a plausible target for the eradication of more aggressive cancer cell subpopulations.

## BACKGROUND

Harboring heterogeneous cell populations in a tumor mass is a common feature of many cancer types [1]. The intratumoral heterogeneity refers to a hierarchical organization of phenotypically and functionally distinct cancer cells within a tumor [2]. Cancer cell subpopulations with distinct biological properties are considered to be the main obstacle against effective cancer therapy [3]. Clonal evolution has been proposed to explain the development of intratumoral heterogeneity [1]. Recently, it has been reported that epigenetic modifications contribute to the genesis of tumor cell heterogeneity [4–6].

MicroRNAs (miRNAs) are a class of non-coding small RNAs that post-transcriptionally regulate gene expression. They pair with the 3′-untranslated regions or the open reading frames of their target mRNAs. The interaction between miRNA and target gene leads to either degradation of the target genes or inhibition of the protein translation [7]. Dysregulation of miRNAs has been implicated in cancer progression by acting as either oncogenes or tumor suppressor genes [8].

Human malignant mesothelioma (HMM) is an aggressive cancer arising from the surface of body cavities [9]. Many factors including environmental contaminants and viruses have been implicated in the development of HMM [10]. Although significant progress has been made in terms of etiology and pathogenesis, the prognosis of HMM patients remains dismal with the survival time of less than 12 months from the diagnosis [9, 10]. HMMs are markedly heterogeneous in morphology as well as in biology, which makes HMM a paradigmatic model for the intratumoral heterogeneity [11]. Formation of three different histopathologic subtypes from single cell *in vitro* indicated the potency of HMM cells in generating tumor heterogeneity [11–13]. Using side population (SP) assay, subpopulation cells harboring stem cell properties were identified in HMM cell lines [13].

In an attempt to elucidate the involvement of epigenetics in the generation and maintenance of intratumoral heterogeneity, the miRNA expression profiles of HMM cell subpopulations were investigated using microarray analysis. A distinct subset of the miRNAs was identified from cancer cell subpopulations, and potential signaling pathways regulated by these miRNAs were determined. The present study provides background information to advance our understanding about the regulatory function of miRNAs in the generation of intratumoral heterogeneity.

## RESULTS

### Isolation of RNA from the sorted SP and NSP cells of MS1

**Figure 1:**
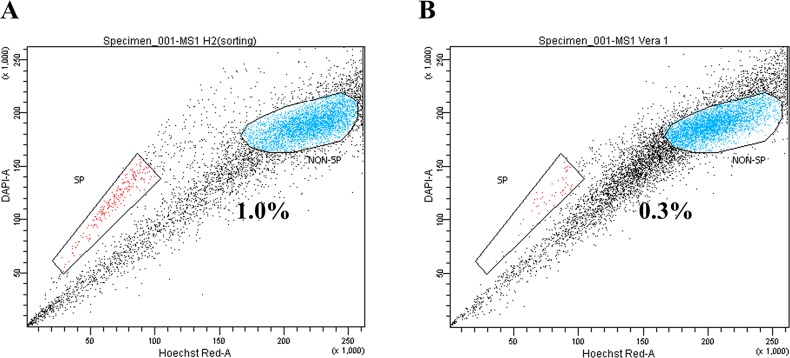
Identification of side population (SP) cells in the MS1 cell line **(A)** Side population assay revealed that the MS1 cell line contained a distinct region of SP cells indicated by a trapezoid on each panel. **(B)** Treatment with verapamil hydrochloride significantly reduced the fraction of SP cells.

SP assay composed of Hoechst 33342 dye staining and subsequent flow cytometry illustrated a distinct SP cells in the MS1 cell line as a tail in flow cytometry plot (Figure 1A). The SP fraction was significantly decreased by the treatment of 50 μM verapamil hydrochloride (Figure 1B). The isolated RNAs were determined to be of good quality with no degradation by A260/280 ratio greater than 1.8 determined using Agilent's 2100 Bioanalyzer and the RNA Integrity Number (RIN) value higher than 8 measured using Nanodrop.

### Identification of differentially expressed miRNAs (DEMs) by microarray analysis

Based on the criteria of over 2-fold difference and p-value less than 0.05, a total of 95 DEMs were identified to be differentially expressed in SP cells compared to NSP cells. Among the 95 DEMs, 42 DEMs were significantly up-regulated and 53 DEMs were significantly down-regulated in the SP cells compared to the NSP cells (Supplementary Table 1). Top 10 up-regulated and down-regulated miRNAs were presented, respectively (Table 1) The microarray data are available at the National Center for Biotechnology Information Gene Expression Omnibus (http://www.ncbi.nlm.nih.gov/geo/) under the accession number of GSE69910.

**Table 1:** Top 10 up- and down-regulated miRNAs defining SP cells identified by miRNA microarray

microRNA	Fold change (log_2_ ratio)	p-value
hsa-mir-3198-1	4.129	2.19E-10
hsa-mir-3198-2	4.129	2.19E-10
hsa-mir-4497	2.666	8.72E-16
hsa-mir-138-1	2.420	2.90E-15
hsa-mir-4304	2.413	1.71E-08
hsa-mir-1281	1.882	3.12E-08
hsa-mir-489	1.859	4.83E-07
hsa-mir-4745	1.850	1.63E-14
hsa-mir-301a	1.782	1.54E-09
hsa-mir-3935	1.642	9.57E-09
hsa-mir-148b	−2.231	1.42E-06
hsa-mir-484	−2.288	0.000582039
hsa-mir-584	−2.290	0.007460966
hsa-mir-425	−2.290	0.0332853
hsa-mir-197	−2.432	4.96E-07
hsa-mir-629	−2.646	0.01597814
hsa-mir-183	−2.835	0.01547796
hsa-mir-4485	−2.884	1.43E-07
hsa-mir-4443	−3.221	2.84E-06
hsa-mir-1246	−4.673	0.000618761

A total of 20 miRNAs that were most significantly altered defining SP cells in MS1 cell line were presented.

### GO and pathway analyses of the predicted target genes of DEMs

**Figure 2:**
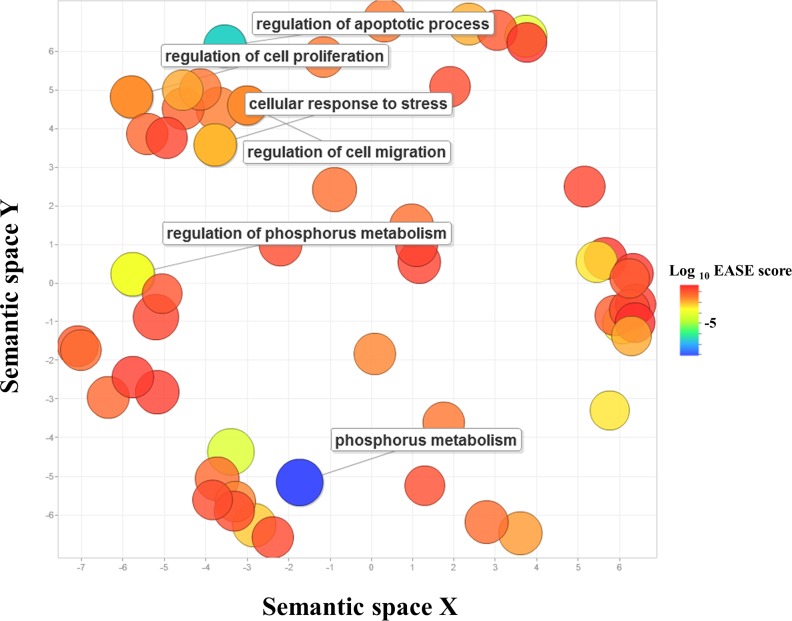
Gene Ontology scatterplot constructed by REVIGO GO terms enriched in predicted target genes of DEMs in SP cells were visualized using REVIGO, which allows to remove functionally redundant GO terms. Individual circle indicates cluster representatives. The color of circle indicates the EASE score. More functionally similar GO terms were closer in the scatterplot, but the semantic space units have no intrinsic meaning. GO terms particularly relevant to more aggressiveness of SP cells were labeled. The full list of GO terms is presented in Supplementary Table 2.

The identification of target genes for miRNAs was performed by several computational algorithms. The TargetScan database was used to predict target genes of DEMs. A total of 1,743 target genes generated by the target prediction software were subjected to the GO analysis in order to determine key-regulatory components and functional relationships of the predicted target genes [14]. The predicted target genes were categorized into biological processes, molecular functions, and cellular components. GO analysis revealed that 287 GO terms were involved in the domain of biological processes including 57 GO terms in the domain of molecular functions and 52 GO terms in the domain of cellular components. To minimize redundancy in the lists of enriched GO categories, the GO terms were merged and replaced with their representative subset of terms based on semantic similarity measures using REVIGO. The initial list of GO terms of biological processes was reduced to 123, eliminating 164 largely redundant terms. The GO terms of 57 molecular functions and 52 cellular components were reduced to 48 and 39 non-redundant terms, respectively. The non-redundant GO terms with higher than 1% frequency were visualized based on their semantic similarities in a semantic space (Figure 2). The cluster representatives of biological processes included regulation of cell proliferation, negative regulation of gene expression, regulation of cell migration, regulation of cellular response to stress, regulation of apoptotic process, and regulation of cell communication. The detailed information about the non-redundant GO terms including frequency, EASE score, and uniqueness, was presented (Supplementary Table 2).

**Figure 3:**
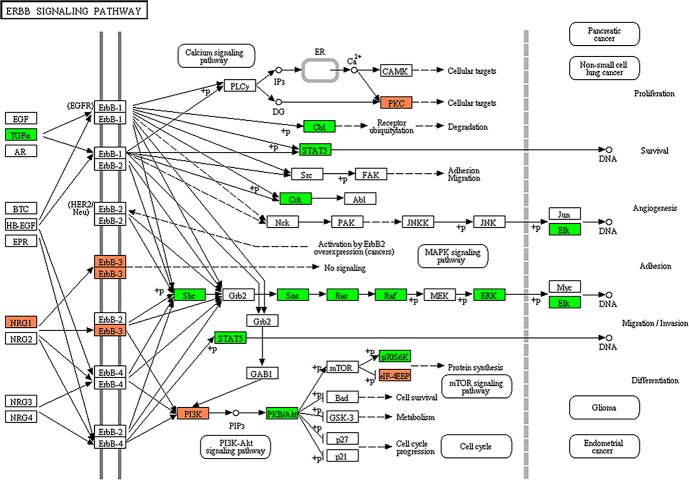
Target prediction of miRNAs and KEGG pathway analysis The ErbB signaling pathway was the highest over-represented pathway among key pathways. Colors represent miRNA target genes and their expression status in KEGG pathway map. Red square: up-regulated target genes. Green square: down-regulated target genes.

The predicted targets of DEMs were subjected to the KEGG pathway annotation to elucidate signaling networks involved in the maintenance of subpopulations of the HMM cells. The functional analysis using KEGG revealed 37 signal transduction pathways significantly involved in the SP cells compared to NSP cells (Supplementary Table 3). One of the most over-represented pathways was ErbB signaling pathway (Figure 3), and some of the other key pathways involved in the tumorigenesis of HMM included MAPK, Wnt, insulin, mTOR, and VEGF signaling pathways.

### Identification of differentially expressed genes (DEGs) by RNA-seq

The NGS data of the transcriptome in SP and NSP cells in the MS1 cell line was previously published [15]. By comparing the RNA-seq data of SP and NSP, as a result, the differential expression of 1,130 genes from a total of 17,122 mRNAs was identified. Among these genes, 795 DEGs were significantly up-regulated and 335 DEGs were down-regulated in the SP cells compared to the NSP cells. The NGS data of the transcriptome in the present study are available in the National Center for Biotechnology Information Sequence Read Archive (http://www.ncbi.nlm.nih.gov/Traces/sra/) under the accession number SRR2062223 and SRR2064655.

### Predicted target genes of DEMs matched to mRNAs from RNA-seq analysis

To validate the microarray analysis of miRNAs, RNA-seq data were integrated to the predicted target genes of the DEMs. The RNA-seq data were used for the validation of miRNA's target genes in SP cells in the present study. Of 1,743 predicted target genes of DEMs generated from the TargetScan database, a total of 88 gene pairs of DEM target genes and DEGs were identified. Based on the criterion of 2-fold difference, 8 up-regulated mRNAs were targeted by 3 down-regulated DEMs, and 4 down-regulated mRNAs were targeted by 3 up-regulated DEMs (Table 2). Of note, down-regulated miR-22 and up-regulated miR-138 were found to have significant association of simultaneous inverse expression in their target genes, *DDIT4* (DNA-damage-inducible transcript 4) and *ROCK2* (Rho-associated, coiled-coil containing protein kinase 2), respectively (p-value < 0.05). In GO and KEGG analyses, the *ROCK2* gene was found to implicate cell migration through regulation of actin cytoskeleton organization and Wnt signaling pathway. In the same vein, the *DDIT4* gene was shown to involve cell survival and death through the regulation of programmed cell death and mTOR signaling pathway (Table 3).

**Table 2:** Potential target genes of miRNAs defining SP cells identified by RNA-seq

microRNAs	Log_2_ FC (microarray)	miRNA-target pairs	Log_2_ FC (RNA-seq)^a^	p-value^b^
hsa-mir-7-1	−1.831	hsa-mir-7-1:*FNDC4*	1.693	0.151
		hsa-mir-7-1:*PPIF*	1.586	0.166
		hsa-mir-7-1:*PTAR1*	1.586	0.166
		hsa-mir-7-1:*POLE4*	1.395	0.166
hsa-mir-22	−1.904	hsa-mir-22:*DDIT4*	2.296	0.047
		hsa-mir-22:*FRAT2*	1.965	0.116
		hsa-mir-22:*IRF5*	1.639	0.192
hsa-mir-183	−1.151	hsa-mir-183:*IDH2*	1.258	0.269
hsa-mir-183*	−2.835	hsa-mir-183*:*IDH2*	1.258	0.269
hsa-mir-138-1	2.420	hsa-mir-138-1:*EID1*	−1.076	0.328
		hsa-mir-138-1:*ROCK2*	−5.283	0.001
hsa-mir-663	1.023	hsa-mir-663:*HSPG2*	−1.728	0.182
hsa-mir-195	1.287	hsa-mir-195:*SLC2A3*	−1.994	0.146

Target gene prediction using RNA-seq identified a total of 12 potential target genes, showing epigenetically concomitant changes in the miRNAs and gene expression in SP cells. The expression statuses of the NSP samples were utilized as references for those in the SP samples.

^a, b^ Fold change and p-value were determined by RNA-seq analysis

Abbreviation: FC, fold change

**Table 3:** Putative biological functions and pathways of ROCK2 and DDIT4 targeted by miR-122 and miR-138-1 via GO and KEGG analyses

miRNAs	Targets	GO and KEGG terms	EASE score
hsa-mir-138-1	*ROCK2*	GO:0006793~phosphorus metabolic process	0.000743227
		GO:0006468~protein amino acid phosphorylation	0.001408809
		GO:0016310~phosphorylation	0.003583528
		GO:0051130~positive regulation of cellular component organization	0.002402911
		GO:0033043~regulation of organelle organization	0.039881837
		GO:0007010~cytoskeleton organization	0.013056657
		GO:0030036~actin cytoskeleton organization	0.013837722
		GO:0030029~actin filament-based process	0.016937947
		GO:0007242~intracellular signaling cascade	0.004744225
		KEGG:has04810~Regulation of actin cytoskeleton	0.000105943
		KEGG:has04310~Wnt signaling pathway	0.000224177
hsa-mir-22	*DDIT4*	GO:0012501~programmed cell death	0.009907439
		GO:0008219~cell death	0.003096555
		GO:0006915~apoptosis	0.007165309
		GO:0009968~negative regulation of signal transduction	2.94131E-05
		GO:0010648~negative regulation of cell communication	7.08217E-05
		KEGG:hsa04150~mTOR signaling pathway	0.017825651

Potential functions of miR-122 and miR-138-1 defining SP cells were presented. These miRNAs were shown to be involved in cell migration, survival, and death, via designated target gene regulation. RNA-seq identified statistical significance of *ROCK2* and *DDIT4* expressions in SP cells. miRTasebase revealed that the genes were experimentally verified targets of miR-122 and miR-138-1, respectively.

## DISCUSSION

In the HMM, accruing evidence has emphasized the importance of aberrantly expressed miRNAs in the etiology and pathogenesis of HMM [16–18]. In the present study, microarray analysis for global expression profile of miRNAs revealed DEMs defining SP cells in a HMM cell line. A total of 95 DEMs including 42 up-regulated DEMs and 53 down-regulated DEMs were identified in SP compared to the NSP of a HMM cell line. In many human malignancies including HMM, SP is enriched for the cells with stem-like properties [14]. To the best of our knowledge, this is the first study to report the global expression profile of miRNAs in the subpopulations of HMM cells.

The notion that miRNAs are responsible for the aggressive behavior of HMM could be supported by the DEMs and their enriched GO terms found in the present study. Among the down-regulated DEMs, miR-148b is a signature of SP cells from hepatocellular carcinoma and has been implicated in enhanced metastatic and angiogenic potential of the hepatic cancer stem cells (CSCs) [19]. Similarly, miR-93 inhibits programmed cell death, facilitates cell proliferation, and promotes the colony formation of colonic CSCs [20]. Consistent with the present study, the oncogenic activation of miR-34a in CSCs with respect to cell proliferation, metastasis, drug resistance, and *in vivo* tumorigenicity have been reported [21–23]. Menges *et al*., [24] reported that aberrant expression of miR-34a was essential for the maintenance of CSC population and metastatic potential of malignant mesothelial cells. They showed that CSC populations lacking miR-34a expression were highly heterogenic and invasive in the genetically modified mice harboring *NF2* and *CDKN2A* gene suppression [25]. One of the upregulated DEMs in our study, miR-138, has been reported to enhance cell survival, *in vitro* tumor sphere formation and *in vivo* tumorigenicity glioma [26].

The SP cells of HMM cell lines are more resistant to the chemotherapeutic drugs than the NSP cells [27]. However, the underlying mechanism of the drug resistance is not comprehensively understood. A few miRNAs identified in this study may be attributable to the enhanced drug resistance of SP cells. Up-regulation of miR-125b-2 increases the drug resistance of glioblastoma CSCs through silencing the Bcl-2 family and inhibition of mitochondria-dependent apoptosis [28]. Likewise, hypomethylation and concordant overexpression of miR-663 reduces the drug sensitivity of human breast cancer cells by repressing the expression of heparan sulfate proteoglycan 2 (HSPG2) [29]. The present study also showed that the expression of HSPG2 was suppressed by up-regulation of miR-663 in HMM cells.

Cancer cells cope with stress by stimulating the expression levels of miRNAs and their target genes [30–32]. The GO analysis in the present study showed that approximately 40% of miRNAs defining SP cells were categorized into biological processes of cellular response to stress. Among the DEMs, miR-7-1 and let-7d may be critical with regard to the stress regulation of SP cells in HMM. Both miRNAs are commonly dysregulated in HMM, promoting malignancy through the activation of *EGF*, *PDGFA*, and *RAS* oncogenes [17, 18, 33]. Flies with mutated miR-7 fail to develop their eyes when grown upon temperature fluctuations [34]. The let-7 family that is commonly down-regulated in many tumor types triggers stress phenotypes of tumor cell [35]. Although further studies are warranted for the detailed mechanisms, SP cells may be quite versatile in adapting to hostile microenvironments.

The ErbB signaling was the one of the most over-represented pathways identified by the KEGG pathway mapping of DEMs and their target genes in this study. The data indicate that the ErbB signaling pathway mediated via dysregulated miRNAs may be the key oncogenic activation of HMM. ErbB tyrosine kinase receptors have been associated with cell proliferation, survival, and differentiation in human solid tumors [36]. The activation of EGFR (ErbB1) and ErbB2 increases the long term survival of asbestos-exposed mesothelial cells *in vivo* and dissemination of HMM [37]. Among the multiple signaling pathways recognized in the present study, the ErbB signaling may be significantly involved in self-renewal and/or survival of more aggressive cancer cell subpopulation in HMM.

Integrated analysis of miRTarBase and RNA-seq revealed a number of potential target genes. Among them, *ROCK2* and *DDIT4* were found to be statistically significant targets of miR-138 and miR-22, respectively. The *ROCK2* is involved in cell migration and phosphorus metabolism in SP cells, and reduced expression of *ROCK2* inhibits migratory and invasive properties of cancer cells [38]. The *DDIT4* is transcribed when exposed to cellular stress such as hypoxia and DNA damage, and it enhances cell growth and survival by inhibiting the cascades of the mammalian target of rapamycin complex 1 [39]. Although further studies for functional validation of target genes of DEMs are necessary, the present study indicates that altered expression of miR-138 and miR-22 may be associated with the maintenance of tumor heterogeneity HMM by regulating their target gene expression.

The failure of the first-line therapy and limited availability of second-line treatment options call for an urgent need of novel strategies to improve the prognosis of HMM patients [10]. Studying heterogeneous cancer cell population within HMM cell lines and tissue may elucidate the molecular mechanisms underlying the generation of tumor cells refractory to current therapies [12]. The DEMs identified in the present study could provide valuable information about the regulatory function of miRNAs in the development of intratumoral heterogeneity of HMM, opening an avenue to devise a novel strategy to cope with the malignant disease. The present study, however, is limited by the lack of a validated experiment. Thus, future research is warranted to scrutinize the mechanism by which miRNAs contribute to the tumor aggressiveness and heterogeneity in HMM.

## MATERIALS AND METHODS

### HMM cell line and culture

A HMM cell line, MS1, was kindly provided by Dr. Jablons (University of California San Francisco). The MS1 cells were determined to be free of mycoplasma contamination by using e-Myco Mycoplasma PCR detection kit (e-Myco, iNtRON Biotechnology, Sungnam, Korea). The cells were cultured in conventional RPMI 1640 medium (Mediatech Inc., Manassas, VA, USA) with 10% fetal bovine serum (FBS; Mediatech Inc.) and supplements at 37°C in a humidified atmosphere containing 5% CO_2_, as previously described [13].

### SP assay and cell sorting

A detailed protocol of SP assay composed of Hoechst 33342 dye staining and subsequent flow cytometry analysis was previously described [13]. Briefly, 10^6^ cells/mL in pre-warmed RPMI containing 2% FBS and 10mM HEPES were incubated with Hoechst 33342 dye (5μg/mL; Sigma–Aldrich, St. Louis, MO, USA) for 90 min at 37°C with intermittent mixing. After centrifugation at 480 × g for 5 minute and subsequent PBS washing containing 2 % FBS at 4°C, the incubated cells were subjected to SP analysis. The flow cytometer sorter (Becton-Dickinson FACS Aria III, Becton-Dickinson, San Jose, CA, USA) equipped with Hoechst Blue with a 450/50 broad pass (BP) filter and Hoechst Red with a 675/30 BP filter was used to detect Hoechst 33342 staining. Verapamil hydrochloride (50 μM, Sigma–Aldrich, St. Louis, MO, USA) which blocks Hoechst 33342 dye efflux was used to identify SP fractions. Three biological replicates of SP and NSP cells were pooled and subjected to further analyses.

### RNA isolation

Total RNAs, including miRNAs, were isolated from the sorted SP and NSP cells using miRNeasy extraction kit (Qiagen, Valencia, CA, USA) according to the manufacturer's instructions. The total RNAs extracted from the sorted cells were pooled and subjected to the miRNA array analysis. RNA quality was assessed by Agilent 2100 bioanalyzer (Agilent Technologies, Palo Alto, CA, USA) using the RNA 6000 Nano Chip, and quantity was determined using Nanodrop-1000 Spectrophotometer (Thermo scientific, Wilmington, DE, USA).

### Expression profiling of human microRNAs by microarray chip assay

Total RNAs with high quality isolated from SP and NSP cells were subjected to microarray assay performed at DNA Link Inc., (Songpa-gu, Seoul, The Republic of Korea). Per RNA sample, 1.6 μg was used as an input into the Affymetrix procedure as recommended by manufacturer's protocol (Affymetrix^®^ FlashTag™ Biotin HSR RNA Labeling Kits, cat. no. HSR30FTA; Genisphere, LLC, Hatfield, PA, USA). Briefly, 1.6 μg of total RNA was tailed by poly (A) and end-labeled by FlashTag™ sequence that has biotin using peroxidase-antiperoxidase enzyme and T4 DNA Ligase, respectively. End-labeled miRNA was hybridized to the GeneChip^®^ miRNA 3.0 arrays (Affymetrix Inc., Santa Clara, CA, USA) for 16 hours at 48°C and 60 rpm. After the hybridization, the chips were stained and washed in a GeneChip Fluidics Station 450 (Affymetrix Inc.) and scanned using a GeneChip Array scanner 3000 7G (Affymetrix Inc.). The expression intensity data were extracted from the scanned images using Affymetrix Command Console software version 1.1 and stored as CEL files.

### Data analysis and miRNA target prediction

The intensity values of CEL files were normalized to remove bias between the arrays, using the Robust Multi-array Average (RMA) and Detected Above Background (DABG) algorithm implemented in the Affymetrix Expression Console software (version 1.3.1.) (http://www.affymetrix.com) [40]. The whole normalized data were imported into the programming environment R (version 3.0.2) and overall signal distributions of each array were compared by plotting using tools available from the Bioconductor Project (http://www.bioconductor.org) to check good normalization [41]. After confirming the normalization of the data, DEMs that showed over 2-fold difference between the average signal values of NSP cells and SP cells were selected. Additionally, the normalized data of the selected miRNAs were also imported into the programming environment R for the statistical t-test and genes with p-value less than 0.05 were extracted as candidate DEMs for further investigation [41]. For the candidate DEMs, targets that have more than 95 context score were subjected to computational prediction algorithm TargetScan 6.2 database (http://www.targetscan.org).

### Gene Ontology and KEGG pathways

Predicted targets of the candidate DEMs identified by the TargetScan 6.2 database were functionally annotated and classified based on the gene functions listed in gene ontology (GO) databases including a web-based tool DAVID (the Database for Annotation, Visualization, and Integrated Discovery) (http://david.abcc.ncifcrf.gov) [42]. Fisher's exact test was used to determine statistical significance between the DEMs and those on the GO annotation list. The GO terms scoring EASE value of < 0.05 were considered statistically significant [43]. The redundancy in the resulting set of GO terms were removed by using REVIGO web server (http://revigo.irb.hr) [44]. SimRel as semantic similarity measure was used, and the allowed similarity was medium (0.7) between the enriched GO terms [44].

Subsequently the predicted targets of the candidate DEMs were subjected to Kyoto Encyclopedia of Genes and Genomes (KEGG) analysis, a pathway mapping tool for molecular networks to identify the biological pathways. The KEGG pathways scoring EASE value of < 0.05 were considered statistically significant.

### RNA-seq and identification of DEGs

The construction of RNA library and detailed analysis of the RNA-seq data was previously performed as described [15]. Briefly, total RNAs with high quality from the sorted SP and NSP cells were subjected to Next Generation Sequencing (NGS) assay performed at the DNA Link Incorporation (Songpa-gu, Seoul, The Republic of Korea). Sequencing libraries of mRNAs were prepared using an Illumina TruSeq RNA Prep kit v2 (Illumina Inc, San Diego, CA, USA.) according to the manufacturer's instructions. The quality of the amplified libraries was verified using an Agilent Technologies 2100 Bioanalyzer (Agilent Technologies, Palo Alto, CA, USA). Cluster generation was carried out in the flow cells on the cBot automated cluster generation system (Illumina Inc.), and then the flow cells were loaded on a HiSeq 2000 sequencing system (Illumina Inc.) with 200 bps paired-end reads. The high quality of clean reads was mapped to the hg19 with TopHat (ver. 2.0.9). The Bam file was used as the output to store a list of read alignments and was added to the Cufflinks software package (ver. 2.0.2) to predict transcript structures and compare transcriptome profiles based on the RNA-Seq data [45]. To compare the expression level of a gene across samples, read counts obtained from RNA-seq were normalized as fragments per kilobase of transcript per million mapped fragments (FPKM) [46]. The FPKM was used to identify DEGs in SP and NSP subpopulations, and then the FPKM in each sample was compared and transformed to the Log_2_ ratio (log_2_(number of SP reads) – log_2_(number of NSP reads)). The gene expression of the NSP subpopulation was used as control data for the determination of up- or down-regulated genes in SP cells. Genes with a p-value of < 0.05 and a log_2_-transformed value smaller than -1 or greater than 1 were considered to be statistically significant DEGs.

### Integrated analysis of DEM targets and DEGs

To validate potential targets of the candidate DEMs identified in the study, a combined prediction of miRTarBase (http://mirtarbase.mbc.nctu.edu.tw/) and RNA-seq was carried out. The miRTarBase is a reliable online software program to search experimentally validated miRNA-target interactions (MTIs) [47]. The RNA-seq that is based on deep-sequencing technology provides a transcription profile in cells, tissues, and organisms [48]. The miRNAs-target mRNA pairs predicted from the TargetScan 6.2 database were analyzed with the miRTarBase data in an integrated way to identify the common miRNA-target pairs. Subsequently, the inverse expression of the potential MTIs was investigated using RNA-seq data. Targets showing over 2-fold difference and p-value less than 0.05 between SP and NSP cells were selected.

## SUPPLEMENTARY MATERIALS AND TABLES








