# Supplementary material for: A subset of microRNAs defining the side population of a human malignant mesothelioma cell line
Source: Oncotarget. 2017 Apr 13;8(26):42847–56. doi: 10.18632/oncotarget.17086 (PMC5522110; doi:10.18632/oncotarget.17086)
Supplement: Supplementary file 1 [file oncotarget-08-42847-s001.pdf]

# A subset of microRNAs defining the side population of a human malignant mesothelioma cell line

## SUPPLEMENTARY MATERIALS

**Supplementary Table 1: List of differentially expressed microRNAs with significant different expression detected in SP cells using microRNA microarray.** A total of 95 miRNAs were differentially expressed between the two groups. Expression profiles of miRNAs were determined in two groups, SP and NSP cells. Data were presented according to the expression levels of individual miRNAs.

**See Supplementary File 1**

**Supplementary Table 2: Cluster representatives of over-represented Gene Ontology biological process annotations associated with different expression microRNAs.** GO analysis was performed using DAVID tools and redundancy reduction was carried out by using REVIGO. Frequency indicates the occurrence of GO term in the underlying GO annotation database; dispensability indicates redundancy of GO term; uniqueness indicates the negative of average similarity of a term to all other terms.

**See Supplementary File 2**

**Supplementary Table 3: Over-represented KEGG pathways with differential expression miRNAs using DAVID tool and related miRNAs.**

**See Supplementary File 3**
